# Traffic-Related Air Toxics and Term Low Birth Weight in Los Angeles County, California

**DOI:** 10.1289/ehp.1103408

**Published:** 2011-08-11

**Authors:** Michelle Wilhelm, Jo Kay Ghosh, Jason Su, Myles Cockburn, Michael Jerrett, Beate Ritz

**Affiliations:** 1 Department of Epidemiology, School of Public Health, University of California, Los Angeles, California, USA; 2 Division of Environmental Health Sciences, School of Public Health, University of California–Berkeley, Berkeley, California, USA; 3 Department of Preventive Medicine, Keck School of Medicine, University of Southern California, Los Angeles, California, USA

**Keywords:** air pollution, air toxics, intrauterine growth retardation, low birth weight, traffic

## Abstract

Background: Numerous studies have linked criteria air pollutants with adverse birth outcomes, but there is less information on the importance of specific emission sources, such as traffic, and air toxics.

Objectives: We used three exposure data sources to examine odds of term low birth weight (LBW) in Los Angeles, California, women when exposed to high levels of traffic-related air pollutants during pregnancy.

Methods: We identified term births during 1 June 2004 to 30 March 2006 to women residing within 5 miles of a South Coast Air Quality Management District (SCAQMD) Multiple Air Toxics Exposure Study (MATES III) monitoring station. Pregnancy period average exposures were estimated for air toxics, including polycyclic aromatic hydrocarbons (PAHs), source-specific particulate matter < 2.5 μm in aerodynamic diameter (PM_2.5_) based on a chemical mass balance model, criteria air pollutants from government monitoring data, and land use regression (LUR) model estimates of nitric oxide (NO), nitrogen dioxide (NO_2_) and nitrogen oxides (NO_x_). Associations between these metrics and odds of term LBW (< 2,500 g) were examined using logistic regression.

Results: Odds of term LBW increased approximately 5% per interquartile range increase in entire pregnancy exposures to several correlated traffic pollutants: LUR measures of NO, NO_2_, and NO_x_, elemental carbon, and PM_2.5_ from diesel and gasoline combustion and paved road dust (geological PM_2.5_).

Conclusions: These analyses provide additional evidence of the potential impact of traffic-related air pollution on fetal growth. Particles from traffic sources should be a focus of future studies.

Air pollution has been linked to low birth weight (LBW) and preterm birth, yet there is no consensus on pollutants or sources responsible for these adverse outcomes. In our previous research in the Los Angeles (LA) Air Basin of Southern California, we observed most consistent associations for high levels of carbon monoxide (CO) and particulate matter with aerodynamic diameter < 10 μm (PM_10_) during the third trimester and term LBW (Ritz and Yu 1999; Wilhelm and Ritz 2005). Our combined results for CO and PM_10_, as well as our research associating residential proximity to traffic during pregnancy with odds of term LBW (Wilhelm and Ritz 2003), suggest that traffic exhaust pollutants may be causative agents of interest for fetal development.

Research in urban areas worldwide has also indicated associations between fetal growth restriction and atmospheric levels of CO, nitrogen dioxide (NO_2_, another criteria pollutant indicative of traffic), and particulate matter (Shah and Balkhair 2011). However, two of four studies relying on residential traffic levels to estimate exhaust exposure (Brauer et al. 2008; Genereux et al. 2008; van den Hooven et al. 2009; Zeka et al. 2008) reported null associations. Several studies associated ambient and personal measures of polycyclic aromatic hydrocarbons (PAHs) with reduced fetal growth (Choi et al. 2008; Dejmek et al. 2000; Perera et al. 2003; Vassilev et al. 2001). PAHs are of interest because they are fuel combustion by-products and can be carried into the body by ultrafine particles < 0.1 μm in aerodynamic diameter (UFP), the main size component of particulate matter directly released by on-road vehicles (Sioutas et al. 2005). PAHs may disturb fetal development, possibly through adverse changes in placental transport or through oxidative stress pathways (Jedrychowski et al. 2010; Perera et al. 2004; Šrám et al. 1999).

Ambient air monitoring data are unlikely to capture the greater spatial heterogeneity of pollutants directly emitted from traffic (Zhou and Levy 2007; Zhu et al. 2002). Because personal measurements of UFP, PAHs, and other traffic constituents are too costly and logistically difficult in large, population-based studies, investigators have used modeling techniques to estimate traffic exposures more accurately than simpler roadway proximity measures. Land use regression (LUR) models based on neighborhood-scale pollutant measurements and geographic information systems (GIS) information on pollution sources and meteorology are examples (Jerrett et al. 2007). LUR models are usually based on pollutants more easily measured, for example, nitric oxide (NO), NO_2_, and nitrogen oxides (NO_x_), as proxies for the mix of toxics in traffic exhaust. To date, six epidemiologic studies, all outside of the United States, used LUR modeling techniques to examine traffic impacts on birth outcomes (Aguilera et al. 2009; Ballester et al. 2010; Brauer et al. 2008; Gehring et al. 2011a, 2011b; Slama et al. 2007); four reported positive associations for various measures of reduced fetal growth.

Here we used three different exposure data sources to examine odds of term LBW in Los Angeles, California, women when exposed to high levels of traffic-related air pollutants prenatally. We created pregnancy exposure estimates based on *a*) government monitoring data for criteria pollutants; *b*) LUR prediction surfaces for NO, NO_2_, and NO_x_ we developed for the LA Basin (Su et al. 2009); and *c*) a unique resource of air toxics monitoring data collected during 2004–2006 by the South Coast Air Quality Management District (SCAQMD) as part of the Multiple Air Toxics Exposure Study (MATES III). These latter data include information on atmospheric levels of various traffic-related air toxics, as well as estimates of source contributions to particulate matter < 2.5 μm in aerodynamic diameter (PM_2.5_) levels based on a chemical mass balance (CMB) analysis.

## Materials and Methods

*Study population.* Electronic birth certificate records for all births occurring 1 June 2004 to 30 March 2006 to women residing in LA County, California, were assembled from the California Department of Public Health (*n* = 276,891). We excluded infants with recorded defects (*n* = 14,777), missing gestational ages (*n* = 12,159), implausible gestational ages (< 140 days or > 320 days, *n* = 2,540), implausible birth weights (< 500 g or > 5,000 g, *n* = 371), and nonsingleton pregnancies (*n* = 5,629), leaving 241,415 total births and 220,528 (91.3%) term births (born at ≥ 37 completed weeks of gestation). Although MATES air toxics measurements began on 1 April 2004, we selected births starting 1 June 2004 to ensure available monitoring data covered at least part of pregnancy (i.e., last trimester).

For these 220,528 births, residential addresses at delivery reported on birth certificates were mapped using a custom geocoder (Goldberg et al. 2008) [see Supplemental Material, Table 1, for details (http://dx.doi.org/10.1289/ehp.1103408)]. The geocoded residential locations (*n* = 219,811; 99.7%) were then intersected with locations of seven MATES III monitoring stations in LA County (Supplemental Material, Figure 1), and women living within 5 miles were selected (*n* = 100,938; 45.9%). A 5-mile radius was used to balance the need for a large sample size with an effort to reduce exposure misclassification, assuming air pollution measurements best represent exposures for women living closer to stations. Among the 100,938 term births, 4.0% were geocoded at the ZIP code level and 0.03% (30 subjects) at the city/county subdivision level.

**Table 1:** Demographic characteristics by outcome group and crude ORs (95% CI) for term LBW.*a*

Parameter	Term LBW cases (*N* = 2,321) *n* (%) or mean ± SD	Noncases (*N* = 98,617) *n* (%) or mean ± SD	Crude term LBW OR (95% CI)
Gestational age (days)		273 ± 10.5		278 ± 10.0		—
Birth weight (g)		2,297 ± 225		3,405 ± 425		—
Sex of infant						
Female		1,297 (55.9)		48,578 (49.3)		1.31 (1.20, 1.42)
Male		1,024 (44.1)		50,039 (50.7)		1.00
Maternal age (years)						
< 20		381 (16.4)		11,468 (11.6)		1.56 (1.37, 1.78)
20–24		654 (28.2)		25,200 (25.6)		1.22 (1.09, 1.36)
25–29		563 (24.3)		26,389 (26.8)		1.00
30–34		417 (17.9)		21,809 (22.1)		0.90 (0.79, 1.02)
≥ 35		306 (13.2)		13,748 (13.9)		1.04 (0.91, 1.20)
Missing				3		
Maternal race/ethnicity*b*						
White, Hispanic		1,650 (71.3)		74,548 (75.8)		1.42 (1.20, 1.68)
White, non-Hispanic		113 (4.9)		7,995 (8.1)		1.00
African American		328 (14.2)		7,130 (7.3)		2.93 (2.42, 3.55)
Asian		130 (5.6)		5,334 (5.4)		1.56 (1.23, 1.98)
Other		93 (4.0)		3,348 (3.4)		1.73 (1.34, 2.22)
Missing		7		262		
Maternal education (years)						
≤ 8		371 (16.2)		15,944 (16.3)		0.93 (0.82, 1.04)
9–12		1,374 (59.9)		54,635 (55.8)		1.00
13–15		316 (13.8)		14,867 (15.2)		0.85 (0.75, 0.96)
≥ 16		231 (10.1)		12,469 (12.7)		0.74 (0.64, 0.85)
Missing		29		702		
Parity						
0		1,094 (47.2)		36,747 (37.3)		1.50 (1.39, 1.63)
≥ 1		1,224 (52.8)		61,839 (62.7)		1.00
Missing		3		31		
Prenatal care						
No prenatal care or started after first trimester		285 (12.3)		8,488 (8.6)		1.49 (1.32, 1.69)
Started in first trimester		2,022 (87.7)		89,827 (91.4)		1.00
Missing		14		302		
Maternal birthplace						
United States		983 (42.4)		37,854 (38.4)		1.18 (1.09, 1.28)
Outside United States		1,335 (57.6)		60,694 (61.6)		1.00
Missing		3		69		
Maternal birthplace						
United States		983 (42.4)		37,854 (38.4)		1.00
Mexico		820 (35.4)		40,222 (40.8)		0.79 (0.72, 0.86)
Other outside United States (includes Puerto Rico)		515 (22.2)		20,472 (20.8)		0.97 (0.87, 1.08)
Missing		3		69		
Primary payment for prenatal care						
Private insurance/HMO/prepaid/Blue Cross-Blue Shield		512 (22.6)		28,758 (29.5)		1.00
Medi-Cal, other government programs, self-pay, no care		1,756 (77.4)		68,857 (70.5)		1.43 (1.30, 1.58)
Missing		53		1,002		
Census-based SES index (quintiles)						
Q1		1,643 (70.8)		65,707 (66.6)		1.65 (1.07, 2.55)
Q2		370 (15.9)		17,239 (17.5)		1.42 (0.91, 2.21)
Q3		200 (8.6)		9,674 (9.8)		1.36 (0.87, 2.15)
Q4		87 (3.8)		4,611 (4.7)		1.25 (0.77, 2.01)
Q5		21 (0.9)		1,386 (1.4)		1.00
**a**Includes 100,938 term births during 1 June 2004 to 30 March 2006 to women residing within 5 miles of a MATES air toxics monitoring station. **b**“Other” race category includes Native American/American Indian, Indian, Filipino, Hawaiian, Guamanian, Samoan, Eskimo, Aleut, Pacific Islander, Other (specified). In regression models, nonwhite Hispanics were included in both the Hispanic category and their specified race category (99.3% of Hispanics stated their race as white).						

This research was approved by the University of California Los Angeles Office of the Human Research Protection Program and the California Committee for the Protection of Human Subjects.

*Exposure assessment.* Monitoring station exposure measures. For 10 of 33 air toxics measured by SCAQMD [see Supplemental Material, Table 2 (http://dx.doi.org/10.1289/ehp.1103408)], we extracted 24-hr averages collected every 3 days at MATES III monitoring stations located within 5 miles of each woman’s residence. As part of MATES III, monthly composite PM_2.5_ filter samples were speciated, and the U.S. Environmental Protection Agency CMB receptor model 8.2 was used to estimate diesel and other source contributions to PM_2.5_ levels during the 2-year study period. In the CMB model, the ambient concentration of chemical species *i* is expressed as a linear equation:


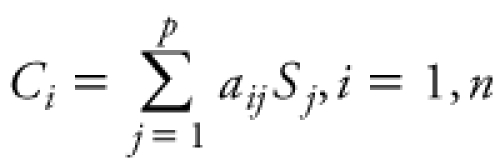
, [1]

**Table 2:** Pollutant distributions for entire pregnancy averages.

Exposure metrics	Pollutant*a*	*n*	Mean ± SD	IQR
LUR_U*b*		NO		100,938		32.2 ± 9.7		10.5
		NO_2_		100,938		26.8 ± 4.1		4.9
		NO_x_		100,938		59.1 ± 12.9		15.1
LUR_S*c*		NO		76,953		34.8 ± 12.6		14.8
		NO_2_		76,953		28.3 ± 4.9		6.4
		NO_x_		76,953		63.5 ± 16.7		20.5
Air toxics		Naphthalene		13,252		188.4 ± 26.8		39.2
		Benzo[*a*]pyrene		13,252		0.13 ± 0.04		0.04
		Benzo[*g,h,i*]perylene		13,252		0.33 ± 0.07		0.11
		Total PAHs		13,252		229.1 ± 30.3		43.3
		Benzene		52,810		0.66 ± 0.13		0.18
		TSP V		55,475		10.5 ± 5.5		4.9
		PM_2.5_ V		55,337		7.5 ± 4.1		3.4
		PM_10_ OC		55,408		5.5 ± 0.58		0.75
		PM_10_ EC		55,408		2.2 ± 0.29		0.41
		PM_25_ OC		55,302		7.4 ± 0.82		1.2
		PM_25_ EC		55,302		1.9 ± 0.29		0.42
		Ammonium nitrate PM_2.5_		64,086		6.2 ± 1.0		1.8
		Ammonium sulfate PM_2.5_		64,086		5.3 ± 1.1		1.4
		Biomass burning PM_2.5_		63,851		0.28 ± 0.10		0.15
		Diesel PM_2.5_		63,941		3.1 ± 0.63		0.83
		Gasoline PM_2.5_		44,481		1.2 ± 0.39		0.61
		Geological PM_2.5_		62,230		1.2 ± 0.41		0.62
		Meat cooking PM_2.5_		51,200		1.6 ± 0.43		0.57
		Residual oil PM_2.5_		64,086		0.54 ± 0.27		0.23
		Sea salt PM_2.5_		64,086		1.5 ± 0.31		0.47
Criteria pollutants		CO		78,161		0.84 ± 0.25		0.37
		NO		77,786		41.2 ± 10.5		14.3
		NO_2_		77,786		29.3 ± 2.9		3.9
		NO_x_		77,786		70.3 ± 12.2		17.0
		O_3_		78,161		34.3 ± 4.7		6.0
		PM_10_		50,020		31.4 ± 3.4		5.4
		PM_2.5_		82,395		17.9 ± 1.8		2.4
**a**Pollutant values are expressed in the following units: NO, NO_2_, NO_x_, O_3_, benzene, ppb; BaP, PAHs, TSP V, vanadium in total suspended particulates; PM_2.5_ V, nanograms per cubic meter; PM_10_, PM_2.5_, PM_10_ OC, PM_10_ EC, PM_2.5_ OC, PM_2.5_ EC, micrograms per cubic meter; CMB estimates for source contributions to PM_2.5_, micrograms per cubic meter; CO, ppm. **b**Unseasonalized LUR model estimates. **c**Seasonalized LUR model estimates.								

where *a_ij_* is the fractional concentration of chemical species *i* in source *j*, *S_j_* is the total mass concentration contributed by source *j*, *p* is the number of sources, and *n* is the number of species, and a least-squares fitting approach is used to estimate *C_i_* (SCAQMD 2008). Monthly average PM_2.5_ concentrations (micrograms per cubic meter) from the following sources were quantified based on the CMB model: diesel exhaust, gasoline exhaust, ammonium nitrate, ammonium sulfate, biomass burning, cooking operations, sea salt, geological (paved road dust), and residual oil burning.

We averaged these data over the following pregnancy periods, relying on the date of birth and gestational age reported on the birth certificate: first trimester (estimated first day of last menstrual period through day 92), second trimester (days 93–185), third trimester (day 186 through birth), and the entire pregnancy. We implemented a 50% completeness criterion to ensure sufficient numbers of daily or monthly readings in each pregnancy averaging period [see Supplemental Material, Table 3, for details (http://dx.doi.org/10.1289/ehp.1103408)].

**Table 3:** Associations between IQR increases in entire pregnancy average air pollution exposures and term LBW.

Crude			Adjusted*a*		
Exposure metric	IQR	*n *(cases/noncases)	OR (95% CI)	*n* (cases/noncases)	OR (95% CI)
NO LUR_U*b*		10.5 ppb		2,321/98,617		1.05 (1.01, 1.10)		2,286/97,764		1.05 (1.00, 1.09)
NO LUR_S*c*		14.8 ppb		1,736/75,217		1.09 (1.03, 1.15)		1,709/74,568		1.08 (1.02, 1.13)
NO_2_ LUR_U		4.9 ppb		2,321/98,617		1.02 (0.97, 1.07)		2,286/97,764		1.03 (0.98, 1.08)
NO_2_ LUR_S		6.4 ppb		1,736/75,217		1.02 (0.96, 1.09)		1,709/74,568		1.04 (0.98, 1.11)
NO_x_ LUR_U		15.1 ppb		2,321/98,617		1.05 (1.00, 1.10)		2,286/97,764		1.04 (1.00, 1.10)
NO_x_ LUR_S		20.5 ppb		1,736/75,217		1.08 (1.02, 1.14)		1,709/74,568		1.07 (1.01, 1.13)
PM_10_ EC		0.41 μg/m^3^		1,248/54,160		1.01 (0.93, 1.09)		1,229/53,675		1.04 (0.96, 1.12)
PM_25_ EC		0.42 μg/m^3^		1,250/54,052		1.05 (0.97, 1.14)		1,231/53,572		1.05 (0.97, 1.14)
Diesel PM_2.5_		0.83 μg/m^3^		1,412/62,529		1.05 (0.98, 1.12)		1,389/61,978		1.06 (0.99, 1.14)
Gasoline PM_2.5_		0.61 μg/m^3^		997/43,484		1.10 (1.00, 1.21)		983/43,153		1.07 (0.97, 1.18)
Geological PM_2.5_		0.62 μg/m^3^		1,365/60,865		1.01 (0.93, 1.10)		1,343/60,327		1.05 (0.97, 1.14)
**a**Adjusted for gestational age (weeks), gestational age (weeks) squared, maternal age, race/ethnicity, education, and parity. **b**Unseasonalized LUR model estimates. **c**Seasonalized LUR model estimates.										

Data from all stations within 5 miles of a woman’s residence that met the completeness criteria were used to generate exposure averages, with inverse-distance weighting of values when more than one station was available. If no stations met the completeness criteria, the value for the exposure period was set to missing.

We also generated criteria pollutant exposure averages, assigning women living near a MATES III station that measured only air toxics to other stations within 5 miles that measured criteria pollutants. Hourly measurements for CO, NO_2_, NO, NO_x_, and ozone (O_3_) (1000–1800 hours) were first averaged over each day if sufficient data were available. These daily averages as well as 24-hr measurements for PM_10_ and PM_2.5_ (collected every 6 and 3 days, respectively) were then averaged over pregnancy periods, again implementing the completeness criteria in Supplemental Material, Table 3 (http://dx.doi.org/10.1289/ehp.1103408). For subjects within 5 miles of more than one station, daily values were inverse distance weighted.

LUR exposure measures. We also extracted NO, NO_2_, and NO_x_ concentration estimates at residential locations from LUR model surfaces we previously developed for the LA Basin (see Su et al. 2009). The LUR surfaces were based on 2-week average Ogawa NO_2_ and NO_x_ measures we collected in September 2006 and February 2007 at 181 locations simultaneously throughout LA County. Final regression models explained 81%, 86%, and 85%, respectively, of the variance in measured NO, NO_2_, and NO_x_ concentrations. Cross-validation analyses suggested high prediction accuracy in the range of 87–91%.

The LUR models most closely approximate annual average concentrations (Su et al. 2009). Thus, in addition to using these unseasonalized estimates, we also created seasonalized LUR measures using government monitoring station measurements nearest to home locations to incorporate yearly and monthly air pollution variations. For example, the LUR estimates for NO were adjusted (multiplied) by the ratio of average ambient NO during each pregnancy month to annual average ambient NO (2006–2007) to generate pregnancy month–specific values. These seasonalized monthly LUR values were then averaged over each pregnancy period. We applied the exclusion criteria described above when generating the pregnancy month scaling factors for NO, NO_2_, and NO_x_, and scaling factors for women within 5 miles of more than one station were based on inverse distance–weighted averages.

*Statistical analyses.* We calculated correlation coefficients and performed a factor analysis (using principal components analysis for initial factor extraction and varimax rotation) to examine clustering among the various air pollution exposure metrics. Associations between air pollution exposure and odds of term LBW (< 2,500 g) were examined using single- and multiple-variable logistic regression models. We calculated odds ratios (ORs) and 95% confidence intervals (CIs) for interquartile range (IQR) and specific unit increases in each exposure metric. We adjusted for maternal age, race/ethnicity, education, and parity (see Table 1 for categories), and gestational age (in weeks) and gestational age squared, as these variables were found to be important confounders in our previous analyses (Ritz and Yu 1999; Wilhelm and Ritz 2003, 2005). We also evaluated changes in OR estimates when additionally controlling for sex of the infant, prenatal care, payment source for prenatal care, whether the mother was born in the United States, maternal birthplace, and a previously developed socioeconomic status (SES) metric (Cheng et al. 2009; Yost et al. 2001). For the SES measure (standardized score for each census block group), principal component analysis was used to develop an index from seven U.S. Census 2000 variables. Because these additional factors did not change air pollution effect estimates by ≥ 5%, they were not included in final models.

## Results

*Characteristics of study populations.* Women residing within 5 miles of MATES III monitoring stations were younger, more likely to be Hispanic, more likely to be born in Mexico, less educated, and much more likely to use Medi-Cal or other government programs versus private insurance for prenatal care compared with the entire population of mothers residing in LA County and delivering infants during the same time period [see Supplemental Material, Table 4 (http://dx.doi.org/10.1289/ehp.1103408)].

The prevalence of term LBW in the study population was 2.1%. In univariate models, odds of term LBW were greater for female infants, firstborn infants, and infants born to younger mothers (< 25 years of age), mothers receiving no prenatal care or receiving care after the first trimester, and to mothers using Medi-Cal or other governmental programs for prenatal care payment (Table 1). Odds of term LBW were lower for infants born to non-Hispanic white mothers and to mothers with more than a high school education (> 12 years). Infants of mothers born in the United States had increased odds of term LBW compared with infants of foreign-born mothers, mostly because of lower odds of term LBW for infants of mothers born in Mexico.

*Exposure metric distributions and correlations.* Entire pregnancy averages. We provide information on distributions of entire pregnancy averages in Table 2 and correlations in Supplemental Material, Table 5 (http://dx.doi.org/10.1289/ehp.1103408). In the factor analysis (Supplemental Material, Table 6), monitoring data–based entire pregnancy averages for the following pollutants clustered most strongly (factor 1): NO, NO_2_, and NO_x_, PM_2.5_, elemental carbon (EC), organic carbon (OC), diesel PM_2.5_, total PAHs (the largest constituent by mass being naphthalene), benzene, biomass burning PM_2.5_, meat cooking PM_2.5_, and ammonium nitrate. A second factor (factor II) represented several pollutants with higher concentrations in the coastal areas (SCAQMD 2008): vanadium, residual oil PM_2.5_, and sea salt PM_2.5_. Vanadium and residual oil PM_2.5_ were strongly positively correlated, as vanadium (along with nickel) was used to identify the residual oil combustion source in the CMB analysis. Other pollutants that clustered positively within this coastal pollutant group were benzo[*a*]pyrene (BaP) and gasoline PM_2.5_, whereas ozone loaded negatively on this factor. Ammonium sulfate loaded positively on factor II, but also on a third factor (factor III), suggesting coastal versus noncoastal spatial patterns were not as strong as for sea salt PM_2.5_, residual oil PM_2.5_, and vanadium. PM_10_ loaded positively on factors I and III, suggesting it does not reflect traffic toxics as strongly as the other pollutants in factor I. Benzo[*g,h,i*]perylene and CO averages loaded similarly on factors I and II, indicating less coastal versus noncoastal differences for these pollutants. All LUR metrics loaded separately on a fourth factor, reflecting their low overall correlation with the monitoring-based exposure metrics (Supplemental Material, Table 5). Finally, geological PM_2.5_ (modeled based on paved road dust samples using iron, calcium, and silica as fitting species) was best represented as a separate fifth factor. Interestingly, gasoline and diesel PM_2.5_ were not strongly correlated spatially in this data set and loaded on separate factors.

Trimester averages. Ambient measures of NO, NO_x_, and total PAHs exhibited strong seasonal variability with peaks in winter, whereas O_3_ and ammonium sulfate followed the opposite pattern with summer peaks. As a result, first- and third-trimester exposures for these pollutants were strongly negatively correlated (*r* = –0.8 to –0.9) (results not shown). Other pollutants with winter peaks but more moderate negative correlations between first- and third-trimester averages (*r* = –0.5 to –0.7) included seasonalized LUR estimates of NO and NO_x_, benzene, EC and OC, and PM_2.5_ from biomass burning, cooking, diesel, and gasoline sources. All other pollutants were less seasonally variable, especially vanadium and residual oil PM_2.5_ (all trimester averages were positively correlated, with *r* = 0.4 to 0.9 and *r* = 0.6 to 0.9, respectively). Second-trimester and entire pregnancy averages for all pollutants were moderately to strongly correlated (*r* = 0.6 to 0.9).

*Associations between exposure metrics and term LBW.* We estimated an approximately 5% increase in adjusted odds of term LBW per IQR increase in entire pregnancy exposures to the following traffic exhaust markers: LUR estimates of NO, NO_2_, and NO_x_ (unseasonalized and seasonalized), EC, and PM_2.5_ from diesel and gasoline combustion (Table 3) [see Supplemental Material, Table 7, for effect estimates based on unit increases (http://dx.doi.org/10.1289/ehp.1103408)]. IQR increases in entire pregnancy exposure to geological PM_2.5_ (representing paved road dust) were also associated with a 5% increase in odds of term LBW. Excluding births that were not geocoded at the parcel, lot, or street level (4.0%) from analyses changed OR estimates by ≤ 0.01. We did not observe associations with IQR increases in entire pregnancy exposures to any of the other pollutants we evaluated (Supplemental Material, Table 7).

For seasonalized LUR estimates of NO, NO_2_, and NO_x_ and ambient measures of CO and PM_2.5_, effect estimates for the first trimester were 1–6% greater than those for the third trimester in models including exposure measures for each trimester (results not shown). For EC and diesel, gasoline and geological PM_2.5_, point estimates were similar, in general, across pregnancy trimesters. However, 95% CIs overlapped widely for all these measures. Overall, the moderate-to-strong negative correlations between first- and third-trimester exposure averages for many pollutants and strong positive correlations between second trimester and entire pregnancy exposure estimates for almost all pollutants limited our ability to identify pregnancy periods exhibiting greater susceptibility.

## Discussion

We estimated an approximately 5% increase in the odds of term LBW per IQR increase in entire pregnancy averages of NO, NO_2_, and NO_x_ as estimated by LUR, EC, and PM_2.5_ from diesel and gasoline exhaust and paved road dust (geological PM_2.5_). The LUR model was built on neighborhood-level measures of NO_x_ and captures small-scale variability in pollutant levels due to proximity to roadway emission sources. The diesel and gasoline exhaust PM_2.5_ averages are direct markers of traffic sources. Although the geological source profile used in the CMB model was based on paved road dust, the association with this PM_2.5_ source further underscores the importance of traffic (i.e., roadways indicator). Finally, EC is often considered a marker of diesel exhaust particles, although there are additional sources of this pollutant in the LA Basin. Altogether, our results for various traffic markers lend strong support to the hypothesis that traffic negatively affects birth weight.

Although diesel PM_2.5_ and EC averages were correlated with total PAH averages (*r* ~ 0.7 to 0.8), we did not observe positive associations between entire pregnancy averages of PAHs and term LBW [Supplemental Material, Table 7 (http://dx.doi.org/10.1289/ehp.1103408)]. These findings might be attributable to the small number of subjects with entire pregnancy PAH averages available and/or the lack of information on spatial variation for these pollutants in our data set, because PAHs were only measured at two stations (West Long Beach and Downtown LA). A larger data set for LA with more information on PAH spatial distributions is required to examine associations further.

Bell et al. (2010) examined associations between birth weight and PM_2.5_ constituents for women residing in Connecticut and Massachusetts and delivering during August 2000 through February 2004. Despite differences in study populations, study design, and constituents used in source apportionment, similarities with our results are notable. In the Northeast, entire pregnancy exposures to constituents most closely associated with motor vehicles, EC and zinc, were associated with lower birth weights and higher risk of term LBW, as were oil combustion–associated elements, vanadium and nickel, and road dust and related constituents such as silicon and aluminum (Bell et al. 2010). In LA County, we also observed positive associations between odds of term LBW and entire pregnancy exposures to EC, as well as diesel, gasoline, and geological PM_2.5_ (paved road dust).

Despite low correlations between LUR and monitoring-based entire pregnancy exposure estimates, we observed positive associations between both metrics and term LBW. This finding suggests spatially heterogeneous local traffic pollution—presumably better represented by our LUR model—as well as regional traffic pollution—represented by the monitoring data—both contribute to increased odds of term LBW. However, both types of metrics may be imperfect markers of the causal pollutants of interest. The LUR models were built on neighborhood-level NO_2_ and NO_x_ concentrations because of the relative ease of measurement. LUR-estimated NO, NO_2_, and NO_x_ have been associated with other health outcomes (Brauer 2010) and are typically considered markers of the suite of pollutants in vehicle exhaust. However, there is limited information on how well spatial and temporal patterns of these pollutants reflect similar patterns for specific toxics of health interest such as PAHs and UFP. Beckerman et al. (2008) reported strong correlations between 1-week average concentrations of NO, NO_2_, and NO_x_ collected by passive monitors at varying distances from a major expressway in Toronto, Canada, and short-term (10-min) measures of UFP (*r* = 0.8 to 0.9). Neighborhood-level monitoring of PAHs, UFP, and other air toxics in conjunction with passive NO, NO_2_, and NO_x_ monitoring in LA is needed to determine whether correlations are similar to those reported for Toronto. In addition, temporal adjustment of LUR pollution surfaces using ambient monitoring station data may not be appropriate because of the unvalidated assumption that ambient monitoring site measures and LUR modeled concentrations co-vary over space. Fewer cases (*n* = 577) had seasonalized versus unseasonalized LUR exposure metrics available, because we relied on ambient monitoring data for temporal adjustment [see Supplemental Material, Table 3, for a description of exclusion criteria for monitoring data (http://dx.doi.org/10.1289/ehp.1103408)]. The unseasonalized LUR measures also best represent spatial, long-term (annual average) exposure contrasts versus pregnancy period–specific exposures. Nonetheless, we observed associations between both unseasonalized and seasonalized LUR measures, suggesting that either local, high-traffic exposures throughout the entire pregnancy are important for term LBW or that our crude seasonalization may not have captured temporal variations in local exposures adequately enough to detect differences across pregnancy periods if they are important.

There is limited animal evidence regarding air pollutants of interest for adverse fetal development. Higher exposure to PAHs during pregnancy measured via personal monitoring, PAH–DNA adducts in umbilical cord blood, and government monitoring stations has been linked to decreased birth weight, length, and head circumference and to small size for gestational age in epidemiologic studies (Choi et al. 2008; Dejmek et al. 2000; Perera et al. 2003, 2004; Vassilev et al. 2001). PAHs may directly affect early trophoblast proliferation, causing suboptimal placentation, reduction in oxygen and nutrient exchange with the fetus, and impairment of fetal growth (Dejmek et al. 2000; Veras et al. 2008).

Oxidative stress may be another biological pathway of interest. UFP in LA have been shown to induce oxidant stress responses and inflammation in experimental studies; these effects may be attributable to the action of PAHs, metals, and related compounds that lead to the production of cytotoxic reactive oxygen species (Cho et al. 2005; Li et al. 2003; Nel et al. 2001; Zhang et al. 1995). Animal and human evidence suggests oxidative stress leads to poor fetal growth (Jedrychowski et al. 2010).

One limitation of this study was the relatively short period (22 months) for which air toxics and speciated PM_2.5_ monitoring data were available, leading to moderate-to-strong negative correlations between first and third trimester measures for many pollutants and strong positive correlations between second trimester and entire pregnancy averages for almost all pollutants. These correlations limited our ability to identify pregnancy periods with greater susceptibility. However, for EC and diesel, gasoline and geological PM_2.5_, we did not detect differences in effect estimates across pregnancy in multitrimester models (results not shown).

We did not observe associations between entire pregnancy averages of CO and PM_2.5_ and odds of term LBW, possibly because of inadequate spatial information for these pollutants (only four of seven MATES III monitors measured criteria pollutants, and for the other monitors we relied on more distant criteria pollutant stations located within 5 miles). In previous studies, we included 4–6 years of births and 12–18 air monitoring stations and thus were able to restrict analyses to women residing within 2 miles of a station (Ritz and Yu 1999; Wilhelm and Ritz 2005). Here we included women residing within 5 miles of a monitoring station to achieve a sufficient sample size, and extrapolating over longer distances likely increased exposure misclassification.

We used the SCAQMD’s MATES III study results to estimate pregnancy exposures to source-specific PM_2.5_ concentrations. SCAQMD (2008) provides a discussion of their data collection and source apportionment modeling methods. Because PM_2.5_ samples were composited for speciation analyses, only monthly average source-specific PM_2.5_ values were available to derive pregnancy averages; thus, temporal variation may not be well represented. The following CMB optimal performance criteria were used by SCAQMD to assess model fit: *R*^2^ values of 0.8–1.0; chi-square values of < 4.0; and differences between calculated and measured PM_2.5_ mass of < 20%. SCAQMD conducted sensitivity tests using various source profiles for diesel and gasoline exhaust, biomass burning, and meat cooking. Final source profiles were selected based on the above performance criteria and comparison of results with previous measurement studies in the LA Basin. For example, the SCAQMD used two separate gasoline source profiles in the CMB model: one developed for the LA Basin as part of the Department of Energy’s Gasoline/Diesel Split Study (Fujita et al. 2006) and a second developed as part of the Northern Front Range Air Quality Study (NFRAQS) (Zielinska et al. 1998). Although there were only minor impacts on other source category estimates, the proportion of PM_2.5_ attributed to gasoline combustion differed substantially depending on which profile was used. Here we used results based on the NFRAQS profile, as recommended in SCAQMD (2008), but acknowledge this as a source of uncertainty in our results for PM_2.5_ attributed to gasoline motor vehicles.

Another source of exposure measurement error is the reliance on address information reported on birth certificates, which does not account for residential mobility during pregnancy. Previous studies indicate that 12–28% of women change residence during pregnancy (Chen et al. 2010; Lupo et al. 2010; Madsen et al. 2010). In our previous population-based survey of women residing in 111 ZIP codes in LA County and delivering in 2003, 22% of women reported moving during pregnancy (Ritz et al. 2007). Air pollution effect estimates based on ambient monitoring data remained unchanged or were slightly strengthened when we restricted analyses to women who did not move. However, smaller spatial-scale exposure estimates (e.g., LUR) may be subject to more error due to residential mobility than regional, background exposure estimates based on monitoring station data.

For this study we relied on information recorded on California birth certificates, limiting our ability to control for certain confounders, such as maternal active or passive smoking. In a previous study incorporating survey data (Ritz et al. 2007), air pollution effect estimates for preterm birth did not change appreciably when we added smoking variables to models already including maternal age, education, race/ethnicity, and parity. Additionally, our population was predominately Hispanic and foreign-born, and prenatal smoking rates among these groups are generally low (< 3%) (California Department of Public Health 2006). Confounding by other SES-related factors potentially correlated with air pollution is also of concern. Our air pollution effect estimates did not change appreciably when we adjusted for prenatal care initiation or payment source or for a census-based SES measure. Also, in our survey-based study, adjustment for family income did not change air pollution effect estimates for preterm birth after adjustment for birth certificate variables (Ritz et al. 2007).

A major strength of our study was the use of novel air toxics and LUR exposure information in addition to routine government monitoring station data for criteria pollutants to help identify specific emission sources of concern for fetal health. Odds of term LBW were positively associated with LUR exposure metrics and PM_2.5_ from diesel and gasoline combustion and paved road dust, suggesting potential importance of mobile source emissions specifically for this outcome. Entire pregnancy averages for CO and PM_2.5_ were correlated with PAH averages, suggesting that these pollutants could have acted as markers for PAHs in previous studies, at least in the LA basin. Ideally, future birth outcome studies would use larger data sets, with neighborhood-level monitoring of PAHs and possibly speciated PM_2.5_ measured in each season.

## Conclusions

These analyses provide additional evidence that local as well as regional traffic-related air pollution adversely impacts fetal growth in Southern California. The positive associations we observed for traffic source particles support previous studies and call for additional research focused on these pollutants. Future birth outcome studies would benefit from measurement and GIS-based models of particle and PAH exposures.

## Correction

A coding error necessitated changes in some of the values in the tables originally published online. These have been corrected here, and do not change the major results or conclusions of the study.

## Supplemental Material

(369 KB) PDF
