# Supplementary material for: Traffic-Related Air Toxics and Term Low Birth Weight in Los Angeles County, California
Source: Environ Health Perspect. 2011 Aug 11;120(1):132–8. doi: 10.1289/ehp.1103408 (PMC3261935; doi:10.1289/ehp.1103408)
Supplement: (369 KB) PDF [file ehp.1103408.s001.pdf]

SUPPLEMENT MATERIAL

Traffic-Related Air Toxics and Term Low Birth Weight in Los Angeles County, California

Michelle Wilhelm[1], Jo Kay Ghosh[1], Jason Su[2], Myles Cockburn[3], Michael Jerrett[2], Beate Ritz[1]

[1] Department of Epidemiology, School of Public Health, University of California, Los Angeles

[2] Division of Environmental Health Sciences, School of Public Health, University of California, Berkeley

[3] Department of Preventive Medicine, Keck School of Medicine, University of Southern California

Supplemental Material, Table 1. Results of birth certificate residential address geocoding.

Mapping level	All term births N, %	Term births within 5 mi of a MATES N, %
Parcel centroid	100,755 (45.7%)	59,470 (58.9%)
Uniform lot interpolation	42,987 (19.5%)	16,406 (16.3%)
Address range interpolation	63,689 (28.9%)	20,983 (20.8%)
Zip code tabulation area centroid, city centroid, or county subdivision centroid	12,380 (5.6%)	4,079 (4.0%)
Ungeocoded	717 (0.3%)	N/A

Supplemental Material, Table 2. List of air toxics included in study.

Total PAHs:<br>naphthalene, benzo[*a*]pyrene, benzo[*a*]anthracene, benzo[*b*+*j*+*k*]fluoranthene, benzo[*g*,*h*,*i*]perylene, indeno(1,2,3-cd)pyrene, dibenzo(a,h)anthracene, chrysene, anthracene, pyrene, fluoranthene, acenaphthylene, acenaphthene, fluorene, and phenanthrene
Naphthalene[a]
Benzo[*a*]pyrene (BaP)
Benzo[*g*,*h*,*i*]perylene (BGP)
Benzene[b]
Toluene
Ethyl benzene
M+p-Xylenes
o-Xylenes
Vanadium

[a] We evaluated naphthalene separately because its levels were on average much higher than those of other PAHs (SCAQMD 2008), benzo[*a*]pyrene separately due to reported health effects in previous studies (Perera et al. 2004) and benzo[*g*,*h*,*i*]perylene separately because of its relatively high correlation with UFP redox activity (Cho et al. 2005).

[b] Benzene, toluene, ethyl benzene and xylenes were selected as VOCs indicative of traffic exhaust (Beckerman et al. 2008). Because all four compounds were highly correlated and produced similar results, we present results for benzene only.

[c] We evaluated the metal vanadium due to the importance of port emissions in the LA Basin (vanadium was used as a marker of residual oil combustion by ships by SCAQMD 2008).

Supplemental Material, Table 3. Exclusion criteria used when estimating pregnancy period specific air pollution exposure metrics.

Pollutant	Criteria
MATES air toxics PAHs[a], BTEX, Vanadium, OC and EC[b]	We required at least 5 available readings for each full pregnancy month in a given period (trimester or entire pregnancy) as well as 5 readings during the last 30 days of a given period.
MATES CMB $PM_{2.5}$ concentrations[b]	The source-specific $PM_{2.5}$ concentration estimates from the CMB analysis were available on a monthly basis (because $PM_{2.5}$ samples had to be composited for speciation). Thus, we took weighted averages of calendar month-specific concentration values based on when pregnancy months occurred. For example, for a woman with a first pregnancy month from January 17 to February 15 (we assumed a 30-day month to simplify calculations), we weighted the corresponding January and February CMB concentration estimates by 50%.<br><br>We also calculated weighted averages of the number of calendar month readings available for each full pregnancy month. For example, for a woman with a first pregnancy month from January 17 to February 15 and with a CMB estimate available for both months, the weighted average number of readings was one. For each full pregnancy month in a given trimester, we required at least 75% of the pregnancy month to fall in calendar month(s) with a CMB source-specific $PM_{2.5}$ value available (i.e., weighted average number of readings >0.75). We also required at least 75% of the last 30 days of pregnancy to fall in calendar month(s) with a CMB reading available.<br><br>We relaxed this criterion for the entire pregnancy period averages since ~15% of monthly values were missing for the gasoline and meat cooking source categories. For entire pregnancy averages, we required at least 75% of all full months during pregnancy to have a CMB value available. For example, for a woman who completed 8 pregnancy months, we required at least 6 monthly CMB readings to be available (based on the sum of the weighted number of readings). We also required at least 50% of the last 30 days of pregnancy to fall in calendar month(s) with a CMB reading available.
CO	Criteria for hourly measurements:<br>At least 50% of hourly values available per 24-hr period and at least 50% of hourly values available from 6am-6pm. If sufficient data were available, a daily (24-hour) average was generated based on the hourly data.<br><br>Criteria for pregnancy periods:<br>We required at least 15 readings for each full month in a given period (trimester or entire pregnancy) as well as 15 readings during the last 30 days of the pregnancy period.
NO, NO_2, NO_x	Criteria for hourly measurements:<br>At least 50% of hourly values available per 24-hr period and at least 50% of hourly values available from 8am-8pm.<br><br>Criteria for pregnancy periods:<br>We required at least 15 readings for each full month in a given period (trimester or entire pregnancy) as well as 15 readings during the last 30 days of the pregnancy period.
O_3 (10am-6pm)	Criteria for hourly measurements:<br>At least 50% of hourly values available from 10am-6pm.<br><br>Criteria for pregnancy periods:<br>We required at least 15 readings for each full month in a given period (trimester or entire pregnancy) as well as 15 readings during the last 30 days of the pregnancy period.
PM_{10}	We required 3 or more values to be available per each full pregnancy month and during the last 30 days of pregnancy.
$PM_{2.5}$[c]	We required 5 or more values to be available per each full pregnancy month and during the last 30 days of pregnancy.

[a] PAH data were only available at the Downtown Los Angeles and West Long Beach stations from 12/8/04-3/30/06. Collection of PAHs was reduced from every 3 days to every 6 days during May-September. Since there were few missing values in the PAH data during the 3 day schedule, we based the exclusion criteria on the every 6 day schedule, i.e., required at least 3 readings for each full month of pregnancy as well as during the last 30 days of pregnancy for each period evaluated. [b] At the Huntington Park station, data were collected from April 2004 through May 2005 only. At Pico Rivera, data were available from April 2004 through May 2005 and August 2005 through March 2006, except source-specific $PM_{2.5}$ concentrations from the CMB model were only available during April 2004 through May 2005. [c] $PM_{2.5}$ measures were available daily at the North Long Beach and Downtown Los Angeles stations.

Supplemental Material, Table 4. Demographic characteristics (n, %) of term LBW cohort compared to all term births in LA County during 6/1/2004 to 3/30/2006.

Parameter	All LA County births (N=220,528)	Geocoded births (N=219,811)	Geocoded births within 5 mi of a MATES station (N=100,938)
**Gestational age (days) (mean ± SD)**	278 ± 10.1	278 ± 10.1	278 ± 10.0
**Birth weight (g) (mean ± SD)**	3384 ± 450	3384 ± 450	3380 ± 453
**Infant gender**			
Female	108792 (49.3)	108442 (49.3)	49875 (49.4)
Male	111736 (50.7)	111369 (50.7)	51063 (50.6)
**Maternal age (years)**			
<20	20819 (9.4)	20757 (9.4)	11849 (11.7)
20-24	48936 (22.2)	48779 (22.2)	25854 (25.6)
25-29	57360 (26.0)	57161 (26.0)	26952 (26.7)
30-34	54799 (24.9)	54633 (24.9)	22226 (22.0)
≥35	38611 (17.5)	38478 (17.5)	14054 (13.9)
Missing	3	3	3
**Maternal race/ethnicity**			
White, Hispanic	137845 (62.8)	137479 (62.8)	76198 (75.7)
White, non-Hispanic	39052 (17.8)	38840 (17.8)	8108 (8.1)
African American	16151 (7.4)	16101 (7.4)	7458 (7.4)
Asian	16885 (7.7)	16845 (7.7)	5464 (5.4)
Other[a]	9589 (4.4)	9552 (4.4)	3441 (3.4)
Missing	1006	994	269
**Maternal education (years)**			
≤8	27241 (12.5)	27165 (12.5)	16315 (16.3)
9-12	104499 (47.9)	104185 (47.9)	56009 (55.9)
13-15	37645 (17.3)	37495 (17.3)	15183 (15.1)
≥16	48641 (22.3)	48484 (22.3)	12700 (12.7)
Missing	2502	2482	731
**Parity**			
0	87448 (39.7)	87126 (39.7)	37841 (37.5)
1 or more	133026 (60.3)	132632 (60.3)	63063 (62.5)
Missing	54	53	34
**Prenatal Care**			
No prenatal care or started after 1st trimester	18258 (8.3)	18163 (8.3)	8773 (8.7)
Started in first trimester	201740 (91.7)	20120 (91.7)	91849 (91.3)
Missing	530	528	316
**Mother's Birthplace (U.S. vs outside U.S.)**			
U.S. Born	96900 (44.0)	96537 (44.0)	38837 (38.5)
Foreign Born	123403 (56.0)	123050 (56.0)	62029 (61.5)
Missing	225	224	72
**Mother's Birthplace**			
U.S.	96900 (44.0)	96537 (44.0)	38837 (38.5)
Mexico	70794 (32.1)	70619 (32.2)	41042 (40.7)
Other outside U.S. (includes Puerto Rico)	52609 (23.9)	52431 (23.9)	20987 (20.8)
Missing	225	224	72
**Primary Payment for Prenatal Care**			
Private insurance/HMO/Pre-paid/Blue Cross-Blue Shield	91298 (42.3)	90990 (42.3)	29270 (29.3)
Medi-Cal, other government programs, self pay, no care	124515 (57.7)	124130 (57.7)	70613 (70.7)
Missing	4715	4691	1055

[a] Includes Native American/American Indian, Indian, Filipino, Hawaiian, Guamanian, Samoan, Eskimo, Aleut, Pacific Islander, Other (specified).

Supplemental Material, Table 5. Pearson's correlation coefficients for entire pregnancy averages.

		Pearson Correlation Coefficients																														
		LUR_U[a]			LUR_S[b]			Air toxics																			Criteria Pollutants					
		NO	NO_2	NO_x	NO	NO_2	NO_x	NAPH	BaP	BGP	PAHS	BENZ	$PM_{2.5}$ V	PM_{10} OC	PM_{10} EC	PM_{25} OC	PM_{25} EC	AN	AS	BB	D	G	GL	MC	RO	SS	CO	NO	NO_2	O_3	PM_{10}	$PM_{2.5}$
**LUR_U[a]**	NO	1.0																														
	NO_2	0.80	1.0																													
	NO_x	0.98	0.84	1.0																												
**LUR_S[b]**	NO	0.82	0.56	0.79	1.0																											
	NO_2	0.70	0.73	0.73	0.72	1.0																										
	NO_x	0.79	0.56	0.80	0.97	0.81	1.0																									
**Air toxics**	NAPH[c]	-0.02	0.23	0.00	0.26	0.18	0.20	1.0																								
	BaP	0.03	-0.36	-0.01	0.48	0.26	0.51	0.29	1.0																							
	BGP	0.01	-0.14	-0.00	0.49	0.29	0.48	0.73	0.85	1.0																						
	PAHS	-0.02	0.20	0.00	0.29	0.20	0.23	1.0	0.33	0.76	1.0																					
	BENZ	-0.13	-0.11	-0.13	0.03	-0.08	0.02	0.87	0.01	0.47	0.85	1.0																				
	$PM_{2.5}$ V[d]	0.09	-0.35	0.05	0.23	0.06	0.28	-0.40	0.68	0.28	-0.34	-0.30	1.0																			
	PM_{10} OC	-0.06	0.03	-0.06	0.13	0.09	0.11	0.85	0.22	0.63	0.88	0.67	-0.36	1.0																		
	PM_{10} EC	0.04	0.02	0.04	0.28	0.22	0.27	0.71	0.58	0.80	0.73	0.38	-0.12	0.71	1.0																	
	PM_{25} OC	-0.01	0.18	0.00	0.05	0.04	0.01	0.71	-0.26	0.21	0.71	0.50	-0.56	0.82	0.67	1.0																
	PM_{25} EC	0.06	0.06	0.06	0.29	0.17	0.27	0.79	0.32	0.67	0.82	0.44	-0.14	0.78	0.83	0.81	1.0															
	AN[d]	-0.02	0.34	0.01	-0.08	0.16	-0.08	0.68	-0.41	0.09	0.67	0.20	-0.71	0.60	0.37	0.73	0.49	1.0														
	AS	0.00	-0.11	-0.00	-0.24	-0.00	-0.16	-0.58	0.36	-0.05	-0.52	-0.24	0.42	-0.16	-0.23	-0.20	-0.12	0.08	1.0													
	BB	0.05	0.04	0.05	0.35	0.18	0.32	0.63	0.43	0.68	0.66	-0.16	0.19	0.20	0.13	0.02	0.26	0.00	-0.49	1.0												
	D	0.06	0.22	0.07	0.23	0.08	0.17	0.72	-0.14	0.31	0.74	0.16	-0.37	0.62	0.49	0.80	0.77	0.43	-0.58	0.56	1.0											
	G	-0.06	-0.27	-0.07	0.14	-0.10	0.14	0.07	0.81	0.63	0.14	0.57	0.22	0.44	0.38	0.30	0.47	-0.09	0.02	-0.19	0.12	1.0										
	GL	-0.06	0.20	-0.05	0.01	0.09	-0.02	0.38	-0.30	-0.05	0.34	0.37	-0.64	0.59	0.43	0.52	0.31	0.55	-0.35	-0.04	0.35	0.15	1.0									
	MC	-0.08	-0.12	-0.08	0.11	-0.03	0.11	0.77	-0.03	0.37	0.73	0.90	-0.17	0.50	0.41	0.40	0.46	0.11	-0.12	-0.26	0.10	0.61	0.26	1.0								
	RO	0.09	-0.34	0.05	0.21	0.06	0.27	-0.40	0.67	0.28	-0.34	-0.31	0.99	-0.33	-0.07	-0.53	-0.08	-0.70	0.35	0.16	-0.33	0.21	-0.67	-0.17	1.0							
	SS	-0.01	-0.15	-0.01	-0.04	0.09	0.03	0.02	0.90	0.67	0.07	0.21	0.36	0.07	0.15	-0.13	0.11	0.10	0.79	-0.39	-0.57	0.23	-0.25	0.35	0.31	1.0						
**Criteria Pollutants**	CO	-0.14	-0.19	-0.14	0.01	-0.28	-0.05	0.42	0.67	0.75	0.49	0.82	-0.13	0.46	0.17	0.34	0.33	0.06	-0.04	-0.23	0.06	0.69	0.19	0.72	-0.18	0.16	1.0					
	NO[e]	-0.07	-0.06	-0.07	0.26	-0.08	0.19	0.89	0.38	0.77	0.91	0.84	-0.26	0.62	0.49	0.47	0.63	0.22	-0.42	0.19	0.43	0.56	0.36	0.80	-0.28	-0.01	0.79	1.0				
	NO_2	-0.06	0.12	-0.05	0.01	0.21	0.04	0.80	-0.18	0.32	0.79	0.72	-0.55	0.58	0.36	0.49	0.43	0.61	-0.06	-0.15	0.19	0.25	0.54	0.64	-0.57	0.18	0.48	0.54	1.0			
	O_3	0.01	0.22	0.02	-0.39	0.10	-0.33	-0.29	-0.94	-0.84	-0.33	-0.30	-0.43	0.01	-0.17	0.12	-0.26	0.46	0.38	-0.33	-0.14	-0.55	0.18	-0.44	-0.36	0.05	-0.40	-0.60	0.16	1.0		
	PM_{10}	-0.04	-0.00	-0.05	-0.04	0.24	0.03	0.47	0.22	0.44	0.54	0.26	-0.01	0.68	0.22	0.50	0.43	0.46	0.52	0.04	0.10	0.11	0.11	-0.12	0.02	0.33	0.35	0.01	0.47	0.46	1.0	
	$PM_{2.5}$	0.01	0.17	0.03	0.08	0.16	0.10	0.70	-0.17	0.27	0.72	0.36	-0.43	0.63	0.22	0.63	0.56	0.75	0.24	0.08	0.42	0.04	0.21	0.19	-0.40	0.21	0.42	0.51	0.61	0.04	0.72	1.0

[a] Unseasonalized LUR model estimates.

[b] Seasonalized LUR model estimates.

[c] NAPH = naphthalene; BaP= benzo[*a*]pyrene; BGP= benzo[*g,h,i*]perylene; BENZ= benzene; AN= ammonium nitrate, AS= ammonium sulfate; BB=biomass burning; D=diesel; G=gasoline; GL=geological; MC=meat cooking; RO=residual oil; SS=sea salt.

[d] Correlations for TSP V were similar to those presented for $PM_{2.5}$ V.

[e] Correlations for NO_x were similar to those presented for NO.

Supplemental Material, Table 6. Varimax rotation, principal component factors for entire pregnancy air pollution averages.[a]

Variable	Factor Loadings					Communality (h_i^2)	Specificity (u_i^2)
	F1	F2	F3	F4	F5		
**First Factor**							
NO_x	**0.99**	0.01	-0.07	0.07	-0.06	0.99	0.01
NO	**0.98**	0.12	-0.09	0.08	-0.08	0.99	0.01
PM_{10} OC	**0.97**	0.05	0.12	0.07	0.16	0.99	0.01
$PM_{2.5}$ EC	**0.97**	0.11	0.04	0.10	0.03	0.97	0.03
Total PAHs	**0.94**	0.13	-0.05	0.07	0.24	0.97	0.03
Naphthalene	**0.94**	0.09	-0.11	0.07	0.25	0.97	0.03
Diesel $PM_{2.5}$	**0.94**	-0.30	0.08	0.06	-0.01	0.98	0.02
NO_2	**0.93**	-0.35	0.03	0.04	0.00	0.98	0.02
Benzene	**0.92**	-0.22	-0.21	0.05	0.17	0.97	0.03
$PM_{2.5}$	**0.92**	-0.28	0.24	0.05	0.06	0.98	0.02
$PM_{2.5}$ OC	**0.91**	-0.41	0.00	0.04	0.02	0.99	0.01
Biomass burning $PM_{2.5}$	**0.90**	0.24	-0.10	0.08	-0.26	0.95	0.05
Ammonium nitrate $PM_{2.5}$	**0.86**	-0.49	0.10	0.02	0.05	0.99	0.01
PM_{10} EC	**0.85**	0.35	-0.21	0.12	0.06	0.91	0.09
PM_{10}	**0.83**	0.20	**0.50**	0.07	-0.02	0.99	0.01
Meat cooking $PM_{2.5}$	**0.78**	-0.32	-0.40	0.03	0.16	0.91	0.09
**Second Factor**							
Sea salt $PM_{2.5}$	-0.06	**0.96**	-0.04	0.07	-0.10	0.95	0.05
Benzo(a)pyrene	0.13	**0.94**	-0.25	0.07	0.06	0.97	0.03
Gasoline $PM_{2.5}$	0.03	**0.93**	0.29	0.08	-0.02	0.97	0.03
O_3 (10am-6pm)	-0.24	**-0.93**	0.13	-0.08	0.20	0.98	0.02
$PM_{2.5}$ vanadium	-0.49	**0.84**	0.18	0.04	-0.12	0.99	0.01
Residual oil $PM_{2.5}$	-0.47	**0.84**	0.17	0.04	-0.18	0.98	0.02
TSP vanadium	-0.50	**0.83**	0.16	0.04	-0.14	0.99	0.01
Benzo(g,h,i)perylene	**0.65**	**0.71**	-0.19	0.09	0.12	0.98	0.02
CO	**0.59**	**0.68**	0.37	0.09	-0.08	0.96	0.04
**Third Factor**							
Ammonium sulfate $PM_{2.5}$	**-0.62**	**0.59**	**0.47**	0.01	-0.03	0.95	0.05
**Fourth Factor**							
NO_x LUR_U[b]	-0.05	-0.09	-0.02	**0.98**	0.01	0.98	0.02
NO LUR_U[b]	-0.08	-0.05	-0.01	**0.97**	0.00	0.95	0.05
NO_2 LUR_S[c]	0.17	0.18	0.05	**0.92**	-0.05	0.91	0.09
NO LUR_S[c]	0.21	0.42	0.00	**0.86**	-0.03	0.96	0.04
NO_x LUR_S[c]	0.15	0.47	0.02	**0.86**	-0.04	0.98	0.02
NO_2 LUR_U[b]	0.23	-0.49	-0.02	**0.79**	0.06	0.93	0.07
**Fifth Factor**							
Geological $PM_{2.5}$	0.22	-0.35	-0.03	-0.04	**0.89**	0.97	0.03
Variance explained	15.55	8.88	1.27	4.97	1.23	$\Sigma h_i^2 = 31.90$	$\Sigma u_i^2 = 1.10$
Percentage of total variance	47.1	26.9	3.9	15.1	3.7	96.7	3.3

[a] Based on 8,293 subjects with non-missing values for entire pregnancy averages for all exposure metrics.
[b] Unseasonalized LUR model estimates.
[c] Seasonalized LUR model estimates.

Supplemental Material, Table 7. Associations between IQR and unit increases in entire pregnancy average air pollution exposures and term LBW.

Exposure Metric	IQR	Crude		Adjusted[a]		Unit increases	Crude	Adjusted[a]
		N (cases, non-cases)	OR (95% CI)	N (cases, non-cases)	OR (95% CI)		OR (95% CI)	OR (95% CI)
NO LUR_U[b]	10.5 ppb	2321, 98617	1.05 (1.01-1.10)	2286, 97764	1.05 (1.00-1.09)	10 ppb	1.05 (1.01-1.10)	1.05 (1.00-1.09)
NO LUR_S[c]	14.8 ppb	1736, 75217	1.09 (1.03-1.15)	1709, 74568	1.08 (1.02-1.13)	10 ppb	1.06 (1.02-1.10)	1.05 (1.01-1.09)
NO_2 LUR_U	4.9 ppb	2321, 98617	1.02 (0.97-1.07)	2286, 97764	1.03 (0.98-1.08)	10 ppb	1.04 (0.94-1.15)	1.05 (0.95-1.17)
NO_2 LUR_S	6.4 ppb	1736, 75217	1.02 (0.96-1.09)	1709, 74568	1.04 (0.98-1.11)	10 ppb	1.04 (0.94-1.14)	1.07 (0.97-1.18)
NO_x LUR_U	15.1 ppb	2321, 98617	1.05 (1.00-1.10)	2286, 97764	1.04 (1.00-1.10)	10 ppb	1.03 (1.00-1.07)	1.03 (1.00-1.06)
NO_x LUR_S	20.5 ppb	1736, 75217	1.08 (1.02-1.14)	1709, 74568	1.07 (1.01-1.13)	10 ppb	1.04 (1.01-1.07)	1.03 (1.00-1.06)
Naphthalene	39.2 ng/m^3	322, 12930	0.93 (0.80-1.10)	312, 12770	0.95 (0.81-1.12)	50 ng/m^3	0.92 (0.75-1.12)	0.94 (0.77-1.15)
Benzo(a)pyrene	0.04 ng/m^3	322, 12930	0.97 (0.87-1.09)	312, 12770	1.00 (0.89-1.12)	0.1 ng/m^3	0.94 (0.72-1.23)	1.00 (0.76-1.15)
Benzo(g,h,i)perylene	0.11 ng/m^3	322, 12930	0.93 (0.78-1.11)	312, 12770	0.97 (0.82-1.16)	0.1 ng/m^3	0.94 (0.79-1.10)	0.98 (0.83-1.15)
Total PAHs	43.3 ng/m^3	322, 12930	0.93 (0.80-1.09)	312, 12770	0.95 (0.81-1.11)	50 ng/m^3	0.92 (0.77-1.10)	0.94 (0.79-1.13)
Benzene[d]	0.18 ppb	1175, 51635	0.97 (0.90-1.05)	1156, 51163	0.95 (0.88-1.03)	0.1 ppb	0.98 (0.94-1.03)	0.97 (0.93-1.02)
PM_{10} OC	0.75 $\mu g/m^3$	1248, 54160	1.00 (0.93-1.08)	1229, 53675	1.02 (0.95-1.10)	1 $\mu g/m^3$	1.00 (0.91-1.11)	1.03 (0.93-1.13)
PM_{10} EC	0.41 $\mu g/m^3$	1248, 54160	1.01 (0.93-1.09)	1229, 53675	1.04 (0.96-1.12)	1 $\mu g/m^3$	1.02 (0.84-1.24)	1.09 (0.90-1.32)
TSP vanadium	4.9 ng/m^3	1253, 54222	1.02 (0.97-1.07)	1234, 53740	1.02 (0.97-1.07)	5 ng/m^3	1.02 (0.97-1.07)	1.02 (0.97-1.07)
PM_{25} OC	1.2 $\mu g/m^3$	1250, 54052	1.02 (0.94-1.11)	1231, 53572	1.03 (0.94-1.12)	0.5 $\mu g/m^3$	1.01 (0.97-1.04)	1.01 (0.98-1.05)
PM_{25} EC	0.42 $\mu g/m^3$	1250, 54052	1.05 (0.97-1.14)	1231, 53572	1.05 (0.97-1.14)	0.5 $\mu g/m^3$	1.06 (0.96-1.17)	1.06 (0.96-1.16)
$PM_{2.5}$ vanadium	3.4 ng/m^3	1250, 54087	1.01 (0.97-1.06)	1231, 53607	1.01 (0.96-1.06)	5 ng/m^3	1.02 (0.95-1.09)	1.02 (0.95-1.09)
Ammonium nitrate $PM_{2.5}$	1.8 $\mu g/m^3$	1421, 62665	0.97 (0.88-1.06)	1398, 62111	0.98 (0.89-1.08)	1 $\mu g/m^3$	0.98 (0.93-1.03)	0.99 (0.94-1.05)
Ammonium sulfate $PM_{2.5}$	1.4 $\mu g/m^3$	1421, 62665	1.00 (0.93-1.07)	1398, 62111	0.98 (0.91-1.05)	1 $\mu g/m^3$	1.00 (0.95-1.05)	0.98 (0.94-1.03)
Biomass burning $PM_{2.5}$	0.15 $\mu g/m^3$	1408, 62443	1.00 (0.93-1.08)	1385, 61893	1.03 (0.96-1.12)	0.1 $\mu g/m^3$	1.00 (0.95-1.06)	1.02 (0.97-1.08)
Diesel $PM_{2.5}$	0.83 $\mu g/m^3$	1412, 62529	1.05 (0.98-1.12)	1389, 61978	1.06 (0.99-1.14)	0.5 $\mu g/m^3$	1.03 (0.99-1.07)	1.04 (1.00-1.08)
Gasoline $PM_{2.5}$	0.61 $\mu g/m^3$	997, 43484	1.10 (1.00-1.21)	983, 43153	1.07 (0.97-1.18)	0.5 $\mu g/m^3$	1.08 (1.00-1.17)	1.06 (0.97-1.14)
Geological $PM_{2.5}$	0.62 $\mu g/m^3$	1365, 60865	1.01 (0.93-1.10)	1343, 60327	1.05 (0.97-1.14)	0.5 $\mu g/m^3$	1.01 (0.95-1.08)	1.04 (0.97-1.11)
Meat cooking $PM_{2.5}$	0.57 $\mu g/m^3$	1162, 50038	1.03 (0.95-1.11)	1143, 49600	0.99 (0.92-1.08)	0.5 $\mu g/m^3$	1.02 (0.96-1.10)	0.99 (0.93-1.07)
Residual oil $PM_{2.5}$	0.23 $\mu g/m^3$	1421, 62665	1.02 (0.98-1.07)	1398, 62111	1.02 (0.97-1.07)	0.1 $\mu g/m^3$	1.01 (0.99-1.03)	1.01 (0.99-1.03)
Sea salt $PM_{2.5}$	0.47 $\mu g/m^3$	1421, 62665	0.95 (0.88-1.03)	1398, 62111	0.94 (0.87-1.02)	0.5 $\mu g/m^3$	0.95 (0.87-1.04)	0.94 (0.86-1.02)
CO	0.37 ppm	1755, 76406	1.03 (0.96-1.10)	1728, 75745	1.00 (0.92-1.07)	0.5 ppm	1.04 (0.94-1.14)	0.99 (0.90-1.10)
NO	14.3 ppb	1749, 76037	0.98 (0.92-1.05)	1722, 75382	0.98 (0.91-1.04)	10 ppb	0.99 (0.94-1.03)	0.98 (0.94-1.03)
NO_2	3.9 ppb	1749, 76037	0.93 (0.88-0.99)	1722, 75382	0.94 (0.88-1.01)	10 ppb	0.84 (0.71-0.98)	0.86 (0.73-1.02)
NO_x	17.0 ppb	1749, 76037	0.97 (0.91-1.04)	1722, 75382	0.97 (0.91-1.03)	10 ppb	0.98 (0.95-1.02)	0.98 (0.94-1.02)
O_3 (10am-6pm)	6.0 ppb	1755, 76406	0.93 (0.88-0.99)	1728, 75745	0.96 (0.90-1.02)	10 ppb	0.89 (0.80-0.99)	0.94 (0.85-1.04)
PM_{10}	5.4 $\mu g/m^3$	1118, 48902	0.94 (0.86-1.03)	1100, 48428	0.98 (0.89-1.08)	10 $\mu g/m^3$	0.89 (0.75-1.06)	0.96 (0.81-1.15)
$PM_{2.5}$	2.4 $\mu g/m^3$	1866, 80529	0.98 (0.92-1.04)	1841, 79848	1.01 (0.95-1.07)	5 $\mu g/m^3$	0.95 (0.84-1.08)	1.01 (0.89-1.15)

[a] Adjusted for gestational age (weeks), gestational age (weeks) squared, maternal age, race/ethnicity and education, and parity. [b] Unseasonalized LUR model estimates. [c] Seasonalized LUR model estimates.

Figure 1. SCAQMD MATES III Monitoring Station Locations

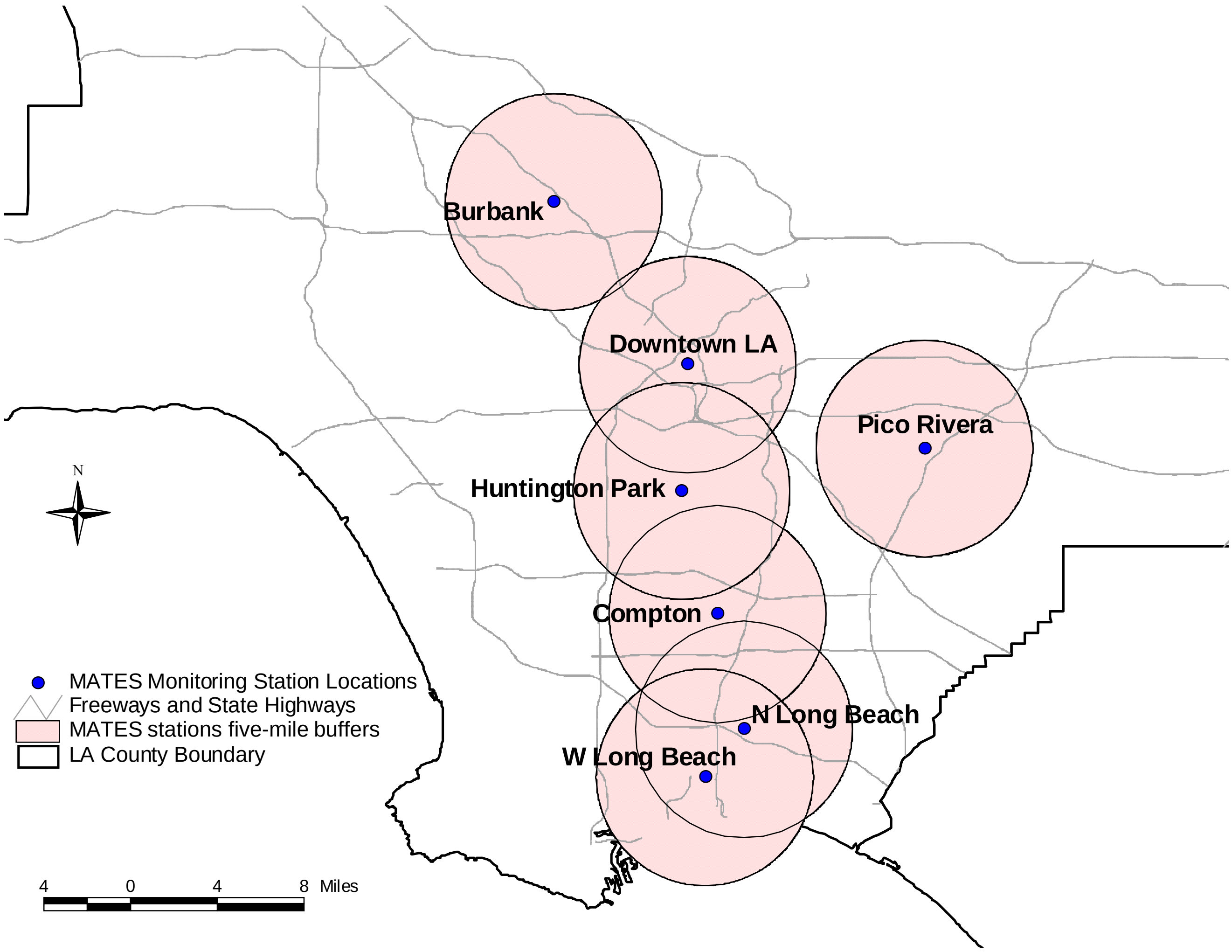

REFERENCES

Beckerman B, Jerrett M, Brook JR, Verma DK, Araine MA, Finkelstein MM. 2008. Correlation of nitrogen dioxide with other traffic pollutants near a major expressway. Atmos Environ 42:275–290.

Cho A, Sioutas C, Miguel AH, Kumagai Y, Schmitz D, Singh M, et al. 2005. Redox activity of airborne particulate matter at different sites in the Los Angeles Basin. Environ Res 99:40-47.

Perera FP, Rauh V, Whyatt RM, Tsai WY, Bernert J, Tu Y, et al. 2004. Molecular evidence of an interaction between prenatal environmental exposures and birth outcomes in a multiethnic population. Environ Health Perspect 112:626-630.

SCAQMD (South Coast Air Quality Management District). 2008. Multiple Air Toxics Exposure Study in the South Coast Air Basin: MATES III Final Report. Diamond Bar, CA: South Coast Air Quality Management District.
